# Genome-wide indel/SSR scanning reveals significant loci associated with excellent agronomic traits of a cabbage (*Brassica oleracea*) elite parental line ‘01–20’

**DOI:** 10.1038/srep41696

**Published:** 2017-02-06

**Authors:** Honghao Lv, Qingbiao Wang, Fengqing Han, Xing Liu, Zhiyuan Fang, Limei Yang, Mu Zhuang, Yumei Liu, Zhansheng Li, Yangyong Zhang

**Affiliations:** 1 Institute of Vegetables and Flowers, Chinese Academy of Agricultural Sciences, Key Laboratory of Biology and Genetic Improvement of Horticultural Crops, Ministry of Agriculture, 12# Zhongguancun Nandajie, Beijing 100081, China; 2 Beijing Vegetable Research Center, Beijing Academy of Agriculture and Forestry Sciences, Key Laboratory of Biology and Genetic Improvement of Horticultural Crops (North China), Ministry of Agriculture, 50# Zhanghua Street, Beijing 100097, China

## Abstract

Elite parental lines are of great significance to crop breeding. To discover unique genomic loci associated with excellent economic traits in the elite cabbage inbred-line ‘01–20’, we performed comparisons of phenotypes as well as whole-genome insertion-deletion/simple sequence repeat loci between ‘01–20’ and each of its five sister lines. ‘01–20’ has a range of excellent agronomic traits, including early-maturing, and improvements in plant type and leaf colour. Eight unique loci were discovered for ‘01–20’ and ‘01-07-258’, another elite line similar to ‘01–20’ at the whole-genome level. In addition, two excellent double-haploid lines derived from a cross of ‘01–20’ also inherited these loci. Based on the quantitative trait locus association results, five of these loci were found to be associated with important agronomic traits, which could explain why the elite parent ‘01–20’ possesses greener outer leaves, a more compact and upright plant-type, rounder head, shorter core length, and better taste. Additionally, some of these loci have clustering effects for quantitative trait loci associated with different traits; therefore, important genes in these regions were analysed. The obtained results should enable marker-assisted multi-trait selection at the whole-genome level in cabbage breeding and provide insights into significant genome loci and their breeding effects.

Elite parental lines, which usually possess a range of excellent properties and high combining ability, are important materials for hybrid breeding^1^. Their use usually results in a batch of excellent hybrid cultivars, which can in turn be used as the basis for new generations of elite parental lines, through combination, optimisation and coordination of genes controlling desirable traits.

With the development of genetics, genomics and molecular biology, elucidating the genetic composition, and its associations with the characteristics, of elite parental lines at the molecular level has become a main approach for improving the accuracy and efficiency of parental selection in plant breeding. At present, studies of elite parents are mainly focused on field crops, such as soybean, barley and maize. Lorenzen *et al*. reported that 80% of the U.S. soybean germplasm could be traced back to just 12 parental lines^2^. In addition, Russell *et al*. reported that 19 elite parental barley lines contained 72% of the genetic variation of modern varieties^3^. Bernardo *et al*. also estimated the parental contribution and coefficient of coancestry of 13 inbred maize lines with known pedigrees using restriction fragment length polymorphism and simple sequence repeat (SSR) markers^4^. Additionally, Kanouni *et al*. studied high yield and good performance using 35 elite chickpea lines^5^. Furthermore, Boudiar *et al*. reported that alleles from the elite Spanish barley cultivar ‘Orria’ contribute to grain yield, reduce plant height and increase 1000-kernel weight, while alleles of chromosome 5H from the landrace ‘SBCC073’ contribute to early vigour, higher grain yield and earlier flowering^6^.

However, few studies on elite parental lines have been reported in vegetable crops. For cabbage, we reported, and performed a pedigree analysis, on the elite inbred line ‘01–20’^7^. We bred this line, together with its five sister lines ‘01-07-258’, ‘01-07-251’, ‘01-1-4’, ‘01–88’ and ‘01-16-5’, through a systemic selection from the variety ‘Early Vikings’ introduced into China from Canada. However, the numbers of F_1_ hybrids resulting from their crosses were 10, 6, 0, 0, 2 and 3, respectively, and the numbers of derived lines (excellent lines selected from their hybrids) were 9, 5, 0, 0, 3 and 0, respectively. To date, some of the hybrids still dominate the market of spring cabbage cultivars in China. The above data show that ‘01–20’ has made a major contribution to cabbage production. Why did ‘01–20’ become an elite parental line, while its sister lines did not? To answer this question, we analysed and compared ‘01–20’ and its five sister lines, as well as two excellent double-haploid (DH) lines derived from ‘01–20’, at the phenotypic and genomic levels, with the objective of identifying the ‘01–20’s distinctive genomic loci associated with excellent agronomic traits.

## Materials and Methods

### Plant materials and field experiment

‘Early Vikings’, a conventional, early-maturing, spring cultivar introduced from Canada in 1966 by the Institute of Vegetables and Flowers, Chinese Academy of Agricultural Sciences (IVF-CAAS), was used in this study as a population with genetic heterogeneity. Genetic heterogeneity was maintained in this population through open self-pollination every year in a greenhouse with honeybees isolated from the outside using a gauze covering.

‘01–20’, an elite parental line, together with its five sister lines: ‘01-07-258’, ‘01-07-251’, ‘01-1-4’, ‘01–88’ and ‘01-16-5’, were all bred through a method of systemic selection from ‘Early Vikings’. ‘01–20’ is an elite, early-maturing, spring cabbage inbred line having upright and green outer leaves, little wax powder, a green and round head, high general and specific combining abilities, and a tender and crisp taste.

As determined in our previous study, the numbers of inbred lines derived from ‘01–20’ and cultivars generated from it had reached 9 and 10, respectively^7^. Table 1 shows some of the varieties generated from ‘01–20’ developed by the Cabbage and Broccoli Breeding Group, IVF-CAAS. Of the 10 generated cultivars, there were two particularly excellent varieties: ‘8398’ and ‘Zhonggan No. 21’. ‘8398’, a cultivar suitable for both protected cultivation and open-field cultivation in the spring, won the second prize at the National Science and Technology Progress Awards of China in 1998. ‘Zhonggan No. 21’, another early-maturing spring cabbage cultivar, also won the same prize in 2014 and, after its introduction in 2006, its cumulative harvesting area had reached over 400,000 ha in China by 2015, making it the top spring cabbage variety in China. These two cultivars still dominate and share over 70% of the spring cabbage market in China.

In addition to the six sister lines, ‘D77’ and ‘D83’, two excellent DH lines, selected from over 200 DH lines derived from the cross between ‘01–20’ and ‘96–100’ (‘Zhonggan No. 18’, see Table 1)^8^, were also used for phenotype measurements and molecular marker assays.

Field trials of the population and the lines mentioned above were carried out at the experimental station of IVF-CAAS in spring 2012 in an open field in Changping District, Beijing. A randomised block design was adopted, with three replications. Each replication/plot consisted of 15 plants. All of the materials were sown on January 27th, transplanted to an open field on March 27th and investigated from mid-May to early June.

### Phenotyping

Observations and measurements of the phenotypes were performed for the main agronomic traits of ‘01–20’, and its five sister lines (‘01-07-258’, ‘01-07-251’, ‘01-1-4’, ‘01–88’ and ‘01-16-5’), two DH lines (‘D77’ and ‘D83’) and the parental population. In total, 25 traits from three categories were assessed (Table 2): plant-type-related traits, including plant diameter (Pd), plant height (Ph) and plant type (Pt) measured by two methods (see Table 2); leaf-related traits, including leaf colour (Lc), leaf number, leaf surface (Ls), leaf wax powder, petiole length (Pl) and petiole width (Pw); head-related traits, including head colour, head maturity period (Hm), head weight (Hw), head vertical diameter (Hvd), head transverse diameter (Htd), core length (Cl), ratio of core length to head vertical diameter (Cl/Hvd), ratio of core width to head transverse diameter (Cw/Htd), head shape index (Hsi), head solidity (Hs), dry matter content (Dmc) and crude fibre content (Cfc); and the trait of seed size (Ss).

These traits were assessed following standards described in ‘Descriptors and data standards for cabbage’^9^ at the rosette or head harvesting stage (Table 2). In addition, Dmc and Cfc were determined following drying and acid, or alkali, digestion methods, respectively, in accordance with the Association of Official Analytical Chemists (AOAC) standards^10^ (Table 2). For colour-related traits, a CR-400 colour difference meter (Konica Minolta, Shanghai, China) was used to assay the leaf and head colour coordinates a* (redness to greenness), b* (yellowness to blueness) and L (lightness to darkness) (CIE1976_Lab standards) with a standard D65 light source, 0° diffuse illumination and a viewing angle of 2° to CIE 1931 under a dark background.

Average values for each trait of each line were calculated from three randomly selected plants in each plot at the rosette or harvesting stage. Adjusted means for the traits were obtained and used for further analysis. Microsoft Excel 2007 (Microsoft, Seattle, WA, USA) and SPSS 12.0 (SPSS, Chicago, IL, USA) software were used for statistical analyses and multiple comparisons.

### Genotyping

Whole-genome scanning for insertion/deletion (indel) and SSR loci was performed for all cabbage materials, using 406 pairs of SSR and indel primers, and the corresponding genetic maps. These markers and maps were developed in our previous study for gene mapping and the detection of quantitative trait loci (QTLs)^8^,^11^, in which we discovered robust QTLs and QTL clusters for 24 main agronomic traits using a DH population of 196 lines derived from a cross of ‘01–20’ × ‘96–100’, indicating that the whole-genome indel/SSR loci and the QTLs were highly reliable.

The molecular marker assay protocol was as follows: polymerase chain reaction (PCR) mixture samples with a volume of 20 μl, which contained 2 μl of PCR buffer (10 ×, Mg^2+^ included), 1.6 μl of dNTP (2.5 mM each), 0.4 μl of Taq DNA polymerase (2.5 U/μl), 5 μl of DNA template (40 ng/μl), 0.6 μl of forward primer (10 μM), 0.6 μl of reverse primer (10 μM) and 9.8 μl of ddH_2_O, were used. The reaction mixture was incubated in a thermal cycler at 94 °C for 5 min, followed by 35 cycles of 94 °C for 30 s, 55 °C for 30 s and 72 °C for 45 s, and finally 72 °C for 7 min. The PCR products were separated on 8% polyacrylamide gels, which were then subjected to silver staining after running at 160 V for 1.5 h^12^. The electrophoresis band patterns for each primer pair was investigated: band pattern the same as ‘01–20’ was recorded as ‘a’, band pattern the same as ‘96–100’ as ‘b’, and a third pattern as ‘c’.

### Associated traits and candidate gene analysis for the distinctive loci

In a previous study, we mapped 144 QTLs for 24 agronomic traits using a DH population of 196 lines derived from a cross between ‘01–20’ and ‘96–100’^13^. Based on the map and QTL information, we determined the positions of the distinctive loci that had been identified in the above molecular marker assays on the map constructed for the QTL analysis, to clarify the association between the traits and these loci. QTLs were named using the following criteria: abbreviation of the trait name, followed by chromosome code and QTL code. For example, *Ph 1.2* represents the second QTL on chromosome C01 associated with plant height.

The genes located in the regions associated with the distinctive loci were analysed and compared with those in *Arabidopsis*, using the annotations for the *Brassica oleracea* reference genome acquired from BRAD (http://brassicadb.org/brad/).

### Equipment and settings

**Figure 1:**
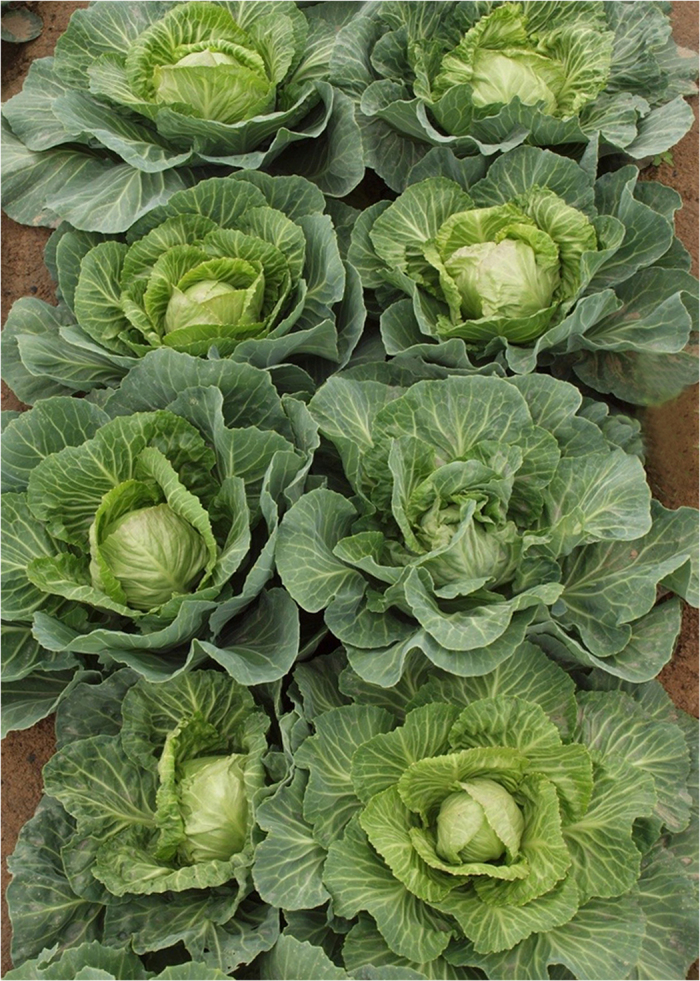
Field Agronomic performance of ‘Early Vikings’, the parental material of 01–20.

**Figure 2:**
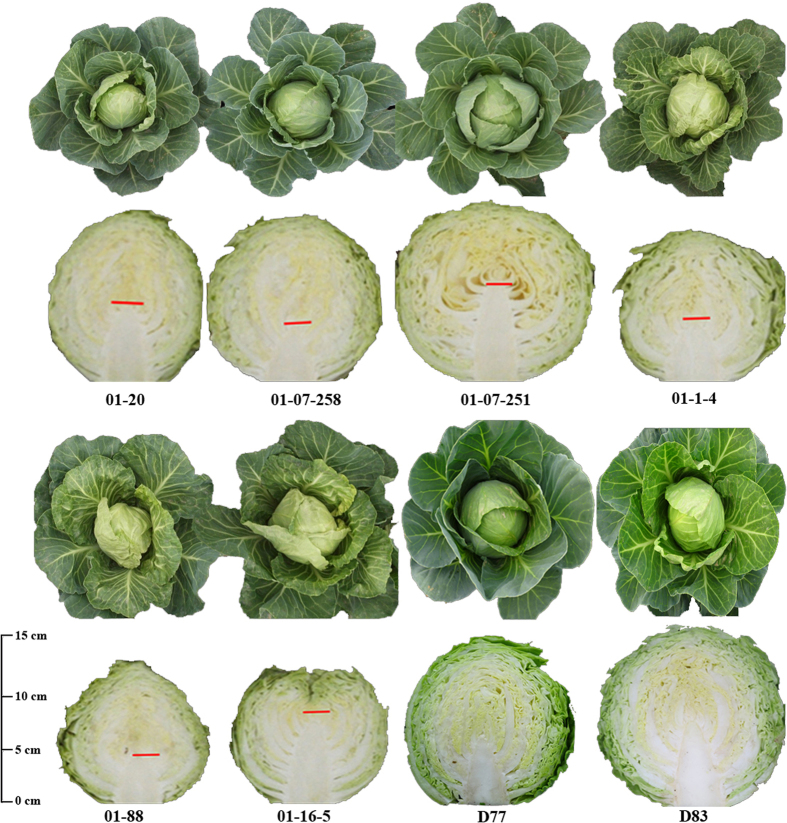
Agronomic Field performance and head traits of 01–20, five of its sister lines, and two DH lines.

**Figure 3:**
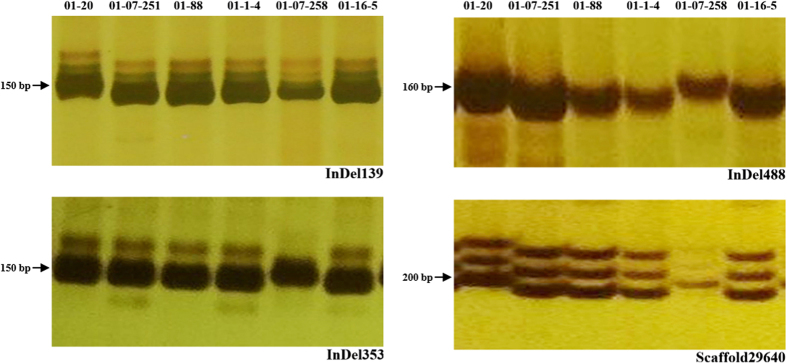
Electrophoresis bands on gels for some polymorphic primers for the six sister lines. This image is from four cropped gels, and full-length gels are presented in Supplementary Figure 2.

**Figure 4:**
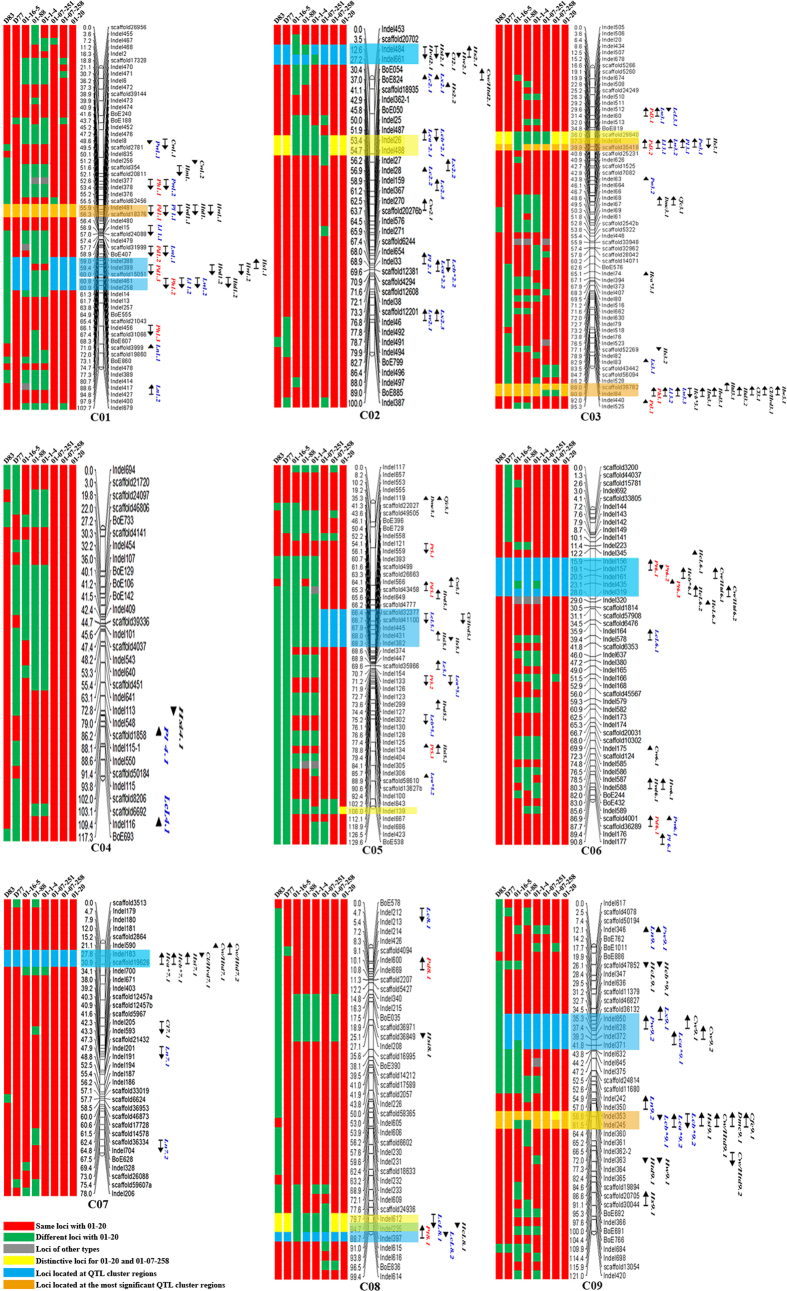
Graphical genotype for 01–20, five of its sister lines, and two DH lines based on whole-genome scanning for SSR/InDelindel/SSR loci Marker locations are listed to the right and recombination distances (cM) to the left of each linkage group. Locations of QTL are indicated by names, bars and arrows to the right of the linkage groups (red: plant type-related traits; blue: leaf-related traits; black: head-related traits). Arrows indicate the relative effect of the 01–20 allele, with downing for positive effects. For abbreviations, see Table 2. Blue blocks on the chromosomes represent QTL cluster regions; orange blocks: the most significant QTL cluster regions; yellow blocks: distinctive loci of 01–20 and 01-07-258.

Images in Figs 1, 2 and 3, were taken using a SONY DSC-HX30 (Sony Co. Ltd., Tokyo, Japan) camera and were edited using Photoshop CS6 software (Adobe Systems Inc., San Jose, CA, USA). Genetic maps in Fig. 4 were generated using JoinMap 4.0 software^14^, and integrated using Photoshop and Microsoft Office 2007 software (Microsoft, Seattle, WA, USA).

## Results

### Clustering analysis of ‘01–20’ and the sister lines

Similarity analyses among the sister lines were conducted using the software NTSYSpc2.11^15^. The loci types (Supplement Table 1) revealed with 406 markers were employed to calculate the genetic similarity coefficients among 9 accessions, ‘01–20’, ‘01-07-258’, ‘01-07-251’, ‘01-1-4’, ‘01–88’, ‘01-16-5’, ‘96–100’, ‘D77’ and ‘D83’. With a genetic similarity coefficient of 0.27, ‘96–100’ was assigned to one cluster, while the remaining eight accessions were assigned to another cluster. The genetic similarity coefficients between ‘01–20’ and the five sister lines, ‘01-07-258’, ‘01-07-251’, ‘01-1-4’, ‘01-16-5’and ‘01–88’, and the two DH lines, ‘D77’ and ‘D83’, were 0.95, 0.93, 0.67, 0.67, 0.65, 0.73 and 0.68 respectively, while the genetic similarity coefficients between ‘96–100’ and ‘D77’ and ‘D83’ were 0.27 and 0.32 respectively. These results indicated that ‘01-07-258’ and ‘01-07-251’ had genetic backgrounds that were closer to ‘01–20’.

### ‘01–20’ shows a range of excellent agronomic traits

Phenotype measurements were carried out for all six sister lines (‘01–20’, ‘01-07-258’, ‘01-07-251’, ‘01-1-4’, ‘01–88’ and ‘01-16-5’), a genetically heterogeneous population compromising 28 individuals (Fig. 1), and the two DH lines. Multiple comparison tests showed that there were significant differences among these materials for most traits, including Pt, Lc, Ls, Cl/Hvd, Hw and Hm. Figure 2 shows the differences in Pt, Hs, Lc and Cl among these lines.

Generally, ‘01-07-258’ was the line most similar to ‘01–20’ in most traits, including Pt, leaf- and head-related traits (Fig. 1; Table 3), and combining ability (from our experience). Thus, ‘01-07-258’ was also deemed as an elite line and used frequently in hybrid breeding. The major difference between the two lines was that the Ss of ‘01-07-258’ (1.42 mm in diameter) was smaller than those of ‘01–20’ (1.80 mm) and the other sister lines.

Among the other sister lines, ‘01-07-251’ had the most upright Pt, the largest plant expansion (Pd and Ph), the highest Hw, the lowest Dmc and Cfc, and the largest Ss, but the longest maturing period (58.67 d) and the longest Cl (8.58 cm); ‘01-1-4’ had the most wrinkled leaf surface and the shortest Hm and Cl, but the smallest head (0.55 kg). ‘01–88’ had the most patulous Pt, a higher Hw and a shorter Hm, but the highest Dmc (6.42) and a low tolerance to splitting; and ‘01-16-5’ had a patulous plant type and greener outer leaves, but the highest Cl/Hvd (0.66) and Cfc (0.61), and the lowest tolerance to splitting. In addition, although ‘Early Vikings’ showed a relatively low uniformity, some individuals still showed outstanding performance in terms of Hm, Hw, leaf and head colour, Hsi and Hs (Fig. 2).

Of the two DH lines, ‘D77’ had the most upright Pt, the greyest Lc and Hc, the largest amount of Lw, the fewest leaves and the longest maturing period. ‘D83’ exhibited the largest plant expansion (Pd and Ph), the highest Hw, and the highest levels of both Dmc and Cfc. Both of these lines have excellent combining ability (data not shown), and thus their use in hybrid seed production is promising.

Thus, ‘01–20’ had a range of excellent agronomic traits and no obvious defects, and it not only inherited the early-maturing trait from ‘Early Vikings’, but also exhibited improvements in Pt, Lc, and head- and quality-associated traits. The DH lines also inherited certain excellent traits from ‘01–20’, such as high Hw, low Cl/Hvd and compact Pt.

### Eight distinctive indel/SSR loci are present in the elite lines

For assays of indel/SSR loci, 406 pairs of SSR and indel primers were applied for the six sister lines, as well as the two DH lines. In total, 398 primer pairs amplified two alleles and eight amplified three alleles at each locus. Overall, 206 primer pairs were polymorphic, accounting for 50.74% of the total. Figure 3 shows some of the polymorphic markers for the six sister lines.

Whole-genome scanning for indel/SSR loci for the six lines revealed that ‘01–20’ had one unique locus, ‘01-07-258’ had one unique locus, and ‘01–20’ and ‘01-07-258’ had six common loci, which were different from those of the other four sister lines (Table 4). In total, 385 out of the 406 loci (accounting for 95%) were the same for ‘01–20’ and ‘01-07-258’, and the loci on C02, C04 and C07 were all the same between the two lines. This is in accordance them being similar lines, and it also indicates that analysing their unique loci together is appropriate.

The distinctive loci were located on chromosomes C02, C03, C05, C08 and C09 (Fig. 4). Some of them clustered together in the same genomic region. For example, the genetic distance between the loci *Indel26* and *Indel488* was shown to be 1.3 cM on C02, and the distance between *scaffold29640* and *Indel64* was 0.7 cM on C03. Figure 4 shows the allele types of all of the distinctive loci for the eight lines.

The seven distinctive loci identified for ‘01–20’ on C02, C03 and C08, were also found in the two excellent DH lines, ‘D77’ and ‘D83”, with the exception of *Indel139* on C05 (Fig. 4), further indicating the significant effects of these loci.

### Five of the distinctive loci are associated with important agronomic traits

The eight newly discovered loci, described above, may help explain why ‘01–20’ and ‘01-07-258’ became elite parental lines. In a previous study^13^, we identified the genomic regions associated with 24 important agronomic traits. Thus, the distinctive loci were anchored to the map published by Lv^13^. We found that five of them, located at the major QTL cluster regions (Fig. 4; indicated in orange: the most significant QTL cluster regions, blue: other major QTL cluster regions) on four chromosomes, were associated with important agronomic traits, including Lc, Hc, Pt, leaf length, Pd and Hs (Table 5). These QTLs were found to explain 6.0–26.1% of the phenotypic variance of these traits.

Several distinctive loci were found to be associated with Lc: *Indel26* and *Indel353* were associated with Lca*, explaining 8.0–9.5% of its phenotypic variance. An analysis of the allele variance effect showed that this allele in ‘01–20’ contributed to a lower trait value than the other alleles. Thus, it had a negative effect (−a to a: green to red), which explains why ‘01–20’ possesses greener leaves (Table 6). *Indel26* was also associated with Lcb*, explaining 12.3% of its phenotypic variance. This allele in ‘01–20’ contributed to a higher trait value than the other alleles. Thus, it had a positive effect (−b to b: blue to yellow), which means that the Lc of ‘01–20’ tends to be yellowish rather than blue (Table 6).

In addition, *Indel235* was also associated with Pt, explaining 11.3% of the phenotypic variance. An analysis of the allele variance effect showed that this allele in ‘01–20’ contributed to a higher trait value than the other alleles. That is, it had a positive effect, which means that ‘01–20’ plants tend to be upright rather than patulous (Table 6).

*Indel64* was identified to be associated with Pd, Ph, Ll, Lw, Pl, Pw and Hs, explaining 6.4–21.2% of the phenotypic variance of these traits. An analysis of the allele variance effect showed that this allele in ‘01–20’ contributed to lower trait values than the other alleles, except for the case of Hs. Thus, it had a negative effect, which explains why the expansion of ‘01–20’ plants is smaller, and the leaves are short and small. Thus, the plants are compact (Table 6).

In addition to Lc, *Indel353*, the distinctive loci for ’01-07-258’ on C09 was also associated with the head quality traits, Dmc and Cfc, Hsi and the core trait Cw/Htd. It can explain 10.1% and 13.9% of the phenotypic variance of Dmc and Cfc, respectively. An analysis of the allele variance effect showed that this allele in ‘01-07-258’ (allele type: b, see Table 6) contributed to higher trait values than the other alleles. Thus, it had a positive effect, which means the taste of ‘01-07-258’ is not as tender and crisp as that of ‘01–20’, which has lower Dmc and Cfc levels (Table 6).

In addition, in a previous study, we identified 12 QTL clustering regions associated with different agronomic traits^13^. Nine of these clusters were possessed by 75% of these lines. These regions may also be key factors in determining whether a line should be maintained for further selection. Thus, the 12 QTL clustering regions on the nine chromosomes may be the selection foundation and these distinctive loci are the core of the foundation.

Thus, the distinctive loci in the elite parental lines are associated with important agronomic traits, which could explain, at the genomic level, why ‘01–20’ has greener leaves, is more compact, has a rounder head and a shorter core length, and tastes better. Loci, like *Indel64* on C03, and the clustering of QTLs for different significant agronomic traits may play particularly important roles in the elite parental line ‘01–20’.

## Discussion

### Application of elite lines: a double-edged sword

Elite lines are of great significance in two ways. First, in plant breeding, they generate a many varieties and contribute greatly to crop production. For example, ‘01–20’, the elite line used in this study has generated as many as 10 varieties, having a spring cabbage market share of over 70%. In rice hybrid production, the elite female parent line ‘Zhenshan’ contributed to a number of widely used rice cultivars in China^16^. Second, they are good materials for studying the genetic effects of significant genomic loci associated with excellent traits, which in turn provide the basis for breeding programs^17^. Using two types of PCR-based DNA markers, Mahatma *et al*. estimated the genetic polymorphisms among nine elite cotton parental lines, which suggested that the genetic constituents of ‘LRA-5166’ are quite different from those of the other eight parental lines^18^. In addition, Lai *et al*. detected more than 1,000,000 single nucleotide polymorphisms, 30,000 indels and 101 low-sequence-diversity chromosomal intervals, as well as hundreds of genes showing presence/absence variation in the maize genome by resequencing six elite maize inbred lines^19^. The current study, for the first time, compared the elite cabbage line ‘01–20’, five sister lines and two derived lines for their phenotypic and genetic constituents, and shed light on the key genomic loci that determined ‘01–20’ as an elite line.

Although elite lines can make major contributions to crop production and quality, excessively applying them may also create problems. During plant breeding, some important agronomic traits, such as disease resistance, high yield and excellent taste, are constantly under directional selection, resulting in significantly reduced genetic diversity^20^. In a study by Hao *et al*. 4% of the 340 wheat base collections from the Northwest Spring Wheat Region in China were found to represent more than 70% of their entire variation^21^. In addition, Ding *et al*. detected a severe reduction in nucleotide variation at *OsAMT1;1*, a high-affinity ammonium transporter in rice (*Oryza sativa*) that controls ammonium uptake capacity, indicating that strong selection on nitrogen uptake-related traits has occurred in rice^22^. Similar reports have been published on soybean 2, barley 3 and wheat^23^,^24^. Additionally, the use of ‘01–20’ as one parent can greatly improve the agronomic traits of hybrids regarding Pt, Lc, Hc, head type, production and taste, among others; however, its lack of disease resistance against *Fusarium* wilt may be risk crop production as this disease is currently affecting cabbage-producing areas in northern China.

### Mining for genes/QTLs associated with important traits using elite parental lines

Elite parental lines are constantly subjected to the directional selection acting on genes that control desirable traits of agronomic importance during their domestication and improvement. Therefore, the genes or loci with a signature of selection from breeders should be identifiable by whole-genome nucleotide polymorphism scanning using DNA markers^25^,^26^.

With the development of molecular biology and of the sequencing of numerous genomes, uncovering the genetic basis of important traits of elite parental lines has become the focus of theoretical and applied studies. One way to characterise genes responsible for phenotypic variation is based on the signature of selection in lines with particularly superior characteristics. Vigouroux *et al*. screened 501 maize genes with a signature of selection and identified 10 as agronomically important candidates because they showed evidence (i.e., the presence of non-neutral SSRs) of exposure to selective pressure. It was further confirmed that one of these, encoding a MADS box transcriptional regulator, experienced a selective sweep during maize domestication^27^. In another study, Yamasaki *et al*. identified eight of the sequenced 1,095 maize genes through a selection test in diverse maize landraces and teosintes, and showed that their functions were consistent with agronomic selection for nutritional quality, maturity and productivity. Another way of identifying genes for corresponding traits is association analysis and QTL mapping using elite lines^28^. For example, Würschum *et al*. detected several QTLs of important traits in rapeseed, including flowering time, Ph, protein content, oil content, glucosinolate content and grain yield^29^. In addition, using genome-wide association mapping, Wang *et al*. identified marker–trait associations in 94 diverse elite wheat lines: marker XwPt-7187 was associated with kernel hardness, XwPt-1250 and XwPt-4628 with test weight, and marker Xgwm512 with Ph^30^.

In the current study, we compared elite line ‘01–20’ with five of its sister lines by scanning SSR/indel loci across the whole genome. We identified eight loci for which the elite lines were distinctive, which were further found to be associated with important agronomic traits, including Lc, Hc, Pt, leaf length, Pd and Hs. To discover interesting candidate genes at these loci, we performed a preliminary analysis for the genes located in the region for the five distinctive loci associated with important agronomic traits, according to the annotations for the *B. oleracea* reference genome acquired from BRAD (gene alignment results, Supplement Table 2). We used the Basic Local Alignment Search Tool (BLAST) (https://www.ncbi.nlm.nih.gov/) tool and set the score cutoff value to 400. The annotation of the functions of the genes included transmembrane transporter, transcription factor, ATP binding, kinase and cytochrome. Based on the alignments with Arabidopsis, some of the genes may be associated with related traits. For example, in Region 2 on chromosome C03, which is associated with Pd, Lw, Ll, Pl, Pw and Hs, the homologous gene ubiquitin ligases *EOL1* can act with *ETO1* and *EOL2* collectively to regulate ethylene biosynthesis in Arabidopsis by controlling type-2 ACC synthase levels^31^. Another homologous gene *SPI* encodes a WD40/BEACH domain protein and shares a similar actin-regulating *ARP2/3* pathway that affects plant growth in various organs^32^. In Region 4 on chromosome C09, which is associated with Lcb*, Lca*, Hsi, Cw/Htd, Dmc and Cfc, the homologous chloroplast-encoded gene *Ycf4* plays an essential role in the Photosystem I complex^33^. These genes may be associated with related traits. However, further studies are still needed to clarify the connection between the candidate genes and the related traits.

The above results shed light on why ‘01–20’, rather than its sister lines, became an elite parental line. These loci could be useful for the development of whole-genome background markers for cabbage breeding and to promote our understanding of the genetic basis of selected traits.

## Additional Information

**How to cite this article:** Lv, H. *et al*. Genome-wide indel/SSR scanning reveals significant loci associated with excellent agronomic traits of a cabbage (*Brassica oleracea*) elite parental line 01-20. *Sci. Rep.* **7**, 41696; doi: 10.1038/srep41696 (2017).

## Supplementary Material

Supplementary Figure 1&2

Supplementary Table 1

Supplementary Table 2

## Figures and Tables

**Table 1:** Some of the derived varieties from the cabbage elite line 01–20.

Variety Name	Planting environment	Pedigree	Certification time	Variety characteristics
8398	Spring open field/protected cultivation	01–20 × 79–156	1998	Extra early-maturing, excellent taste and high production.
Zhonggan No. 15	Spring open field	01–20 × 84–05	1998	Early-maturing, excellent taste and high production.
Zhonggan No. 16	Spring open field	01–20 × 21–3	1999	Late-maturing, excellent taste and high production.
Zhonggan No. 10	Spring open field /protected cultivation	01–20 × 02–12	2002	Extra early-maturing and excellent taste.
Zhonggan No. 18	Spring/autumn open field	01–20 × 96–100	2002	Late-maturing, high production and resistant to black rot and *Fusarium* wilt.
Zhonggan No. 21	Spring open field /protected cultivation	01–20 × 87–534	2006	Early-maturing, excellent taste, high production and tolerant to splitting.
Zhonggan No. 23	Spring open field	01–20 × 88–62	2006	Middle-maturing, high production and resistant to *Fusarium* wilt.
Zhonggan No. 165	Spring open field	01–20 × 10–795	2014	Middle-maturing and high production.

**Table 2:** Designation of traits and description of trait measurements.

Trait	Abb.	Assessment time	Valuation criteria
Plant diameter	Pd	Harvesting stage	The maximum horizontal distance of the rosette leaves (unit: cm). Accurate to 0.1 cm.
Plant height	Ph	Harvesting stage	The distance from the plant top to the ground (unit: cm). Accurate to 0.1 cm.
Plant type 1	Pt1	Rosette stage	Evaluation of the angle between the petiole and the horizontal plane.
Plant type 2	Pt2	Rosette stage	Visual measurement of the angle between the petiole and the horizontal plane: 1: upright; 2: half-upright; 3: half-patulous; 4: patulous.
Leaf colour	Lc, Lca*, Lcb* and LcL	Rosette stage	Method 1: visual measurement of the rosette leaves’ colour from the front: 1: slightly green; 2: green; 3: dark green; 4: slightly grey/green; 5: grey/green; 6: dark grey/green.Method 2: Colour coordinates a*, b* and L were measured using a CR-400 colour difference meter.
Leaf length	Ll	Harvesting stage	The maximum length of the largest leaf (unit: cm). Accurate to 0.1 cm.
Leaf width	Lw	Harvesting stage	The maximum width of the largest leaf (unit: cm). Accurate to 0.1 cm.
Leaf number	Ln	Harvesting stage	The number of leaves left after head harvesting.
Leaf surface	Ls	Rosette stage	Visual measurement of the surface of the rosette leaves: 1: smooth; 2: slightly wrinkled; 3: wrinkled; 4: very wrinkled.
Leaf wax powder	Lx	Rosette stage	Visual measurement of the leaf wax powder: six levels were classified according to the quantity.
Petiole length	Pl	Harvesting stage	The maximum length of the petiole of the largest rosette leaf (unit: cm). Accurate to 0.1 cm.
Petiole width	Pw	Harvesting stage	The maximum width of the basal petiole of the largest rosette leaf (unit: cm). Accurate to 0.1 cm.
Head colour	Hc, Hca*, Hcb*, and HcL	Harvesting stage	Measured using two methods:1. Method 1: visual measurement of the head colour: 1: slightly green; 2: green; 3: dark green; 4: slightly grey/green; 5: grey/green; 6: dark grey/green.2. Colour coordinates a*, b* and L were measured on the top of the head using a CR-400 colour difference meter.
Head mature period	Hm	Harvesting stage	Days from transplanting to harvesting.
Head weight	Hw	Harvesting stage	Weight of a matured cabbage head (unit: kg).
Head vertical diameter	Hvd	Harvesting stage	Height from the base to the top of a matured cabbage head (unit: cm). Accurate to 0.1 cm.
Head transverse diameter	Htd	Harvesting stage	The maximum transverse diameter of a cut-open head from the middle (unit: cm). Accurate to 0.1 cm.
Core length	Cl	Harvesting stage	Length of the core in the head.
Core width	Cw	Harvesting stage	Width of the core base in the head.
Cl/Hvd	Cl/Hvd	Harvesting stage	The ratio of Cl to Hvd.
Cw/Htd	Cw/Htd	Harvesting stage	The ratio of Cw to Htd.
Head shape index	Hsi	Harvesting stage	Hsi = Htd/Hvd.
Head solidity	Hs	Harvesting stage	Hs = Hw/(π/6 * Hvd* Htd^2^).
Dry matter content	Dmc	Harvesting stage	The head was cut open and sliced to 1–2 cm after removing the core and 500 g was randomly sampled and dried to constant weight (M) at 105 °C. Dmc = M/500* 100% (AOAC standards^10^).
Crude fibre content	Cfc	Harvesting stage	The crude fibre content was assayed by acid digestion and alkali digestion (AOAC standards^10^).
Seed size	Ss	Seed	The diameter of a seed.

**Table 3:** Statistical analysis for the main agronomic traits of 01–20, the other lines and Early Vikings.

Trait	01–20	01-07-258	01-07-251	01-1-4	01–88	01-16-5	D77	D83	Early Vikings		
									Average	Min.	Max.
Pd	36.72 ± 1.25b	34.25 ± 1.11c	41.70 ± 0.22a	37.25 ± 0.63b	43.78 ± 0.73a	37.96 ± 1.13b	35.88 ± 1.13b	43.27 ± 1.13a	42.08 ± 0.88a	29.08	58.16
Ph	19.39 ± 1.03c	18.47 ± 0.26d	24.81 ± 0.27a	17.51 ± 0.61d	21.54 ± 0.78b	18.64 ± 0.18d	22.88 ± 0.11b	24.72 ± 0.28a	22.53 ± 0.47b	14.24	35.36
Pt1	57.65 ± 0.50b	55.78 ± 0.53c	59.74 ± 0.49a	46.47 ± 0.33e	43.78 ± 0.74 f	52.40 ± 0.25d	60.08 ± 0.24a	51.57 ± 0.33d	52.33 ± 0.47d	45.50	61.25
Pt2	1e	2d	1e	3b	3b	3b	4a	2d	2.60 ± 0.06c	1	4
Lc	2e	2e	2e	4b	3d	1 f	5a	2e	3.20 ± 0.06c	1	4
Lca*	−11.82 ± 0.05bc	−11.27 ± 0.84b	−12.58 ± 0.18 cd	−13.57 ± 0.02d	−13.75 ± 0.20d	−16.18 ± 0.53e	−10.82 ± 0.38a	−10.73 ± 0.51a	−12.83 ± 0.65cd	−17.14	−10.95
Lcb*	16.72 ± 0.21bc	15.61 ± 0.14d	15.79 ± 0.51 cd	22.03 ± 0.02a	16.90 ± 0.09b	21.99 ± 0.14a	14.09 ± 0.08e	15.61 ± 0.12d	17.39 ± 0.12b	15.39	18.07
LcL	45.56 ± 0.71bc	46.08 ± 1.42bc	43.89 ± 1.49c	46.88 ± 1.61b	47.25 ± 0.28b	49.72 ± 0.18a	45.08 ± 0.15bc	44.28 ± 0.31c	46.65 ± 1.00b	44.26	51.36
Ll	21.03 ± 0.09d	20.77 ± 0.25d	26.83 ± 0.07a	21.33 ± 0.16cd	25.01 ± 0.38b	19.80 ± 0.85e	22.31 ± 0.72c	22.25 ± 0.62c	21.85 ± 0.05c	16.48	27.51
Lw	16.45 ± 0.43bc	16.30 ± 0.67c	18.78 ± 0.02a	17.14 ± 0.27b	19.12 ± 0.40a	17.09 ± 0.82bc	16.62 ± 0.35bc	18.87 ± 0.19a	19.37 ± 0.03a	12.80	24.03
Ln	18.33 ± 0.84c	17.66 ± 0.50c	23.50 ± 1.10a	17.84 ± 0.38c	21.11 ± 0.22b	23.01 ± 1.13a	14.94 ± 0.59d	21.11 ± 0.28b	21.20 ± 1.14b	16	39
Ls	2c	2c	1d	3a	3a	3a	2c	3a	2.19 ± 0.06b	1	3
Lx	2c	2c	2c	1d	1d	1d	6a	2c	2.80 ± 0.09b	1	4
Pl	0e	3.85 ± 0.13c	0e	4.04 ± 0.05b	0e	0e	4.47 ± 0.02a	0e	2.52 ± 0.01d	0	5.21
Pw	2.86 ± 0.01a	2.85 ± 0.11a	2.82 ± 0.03ab	2.78 ± 0.02ab	2.86 ± 0.02a	2.84 ± 0.03a	2.71 ± 0.02c	2.74 ± 0.01bc	2.73 ± 0.09b	2.42	3.04
Hc	2c	2c	1d	1d	1d	1d	5a	2c	3.57 ± 0.03b	1	6
Hca*	−17.16 ± 0.26bc	−17.02 ± 0.66bc	−17.49 ± 0.62c	−16.89 ± 0.17bc	−18.83 ± 0.79d	−16.49 ± 0.56b	−15.96 ± 0.17a	−16.31 ± 0.39b	−17.49 ± 0.16c	−18.95	−16.10
Hcb*	30.01 ± 0.01 cd	29.82 ± 0.85d	29.35 ± 0.21d	32.27 ± 0.87b	35.06 ± 0.31a	31.59 ± 1.77bc	25.72 ± 0.13e	26.21 ± 0.22e	32.32 ± 0.84b	28.86	36.58
HcL	57.57 ± 0.68d	56.90 ± 0.48d	55.69 ± 0.01e	65.30 ± 0.01a	65.32 ± 0.27a	62.46 ± 1.19b	54.85 ± 0.19ef	53.47 ± 0.21f	59.59 ± 0.24c	54.63	66.08
Hm	56a	52.01 ± 0.85ab	58.67 ± 0.67a	50.02 ± 0.58c	52.67 ± 0.33ab	55.67 ± 0.67a	60.01 ± 0.25a	59.05 ± 0.31a	59.67 ± 1.33a	52	71
Hw	0.70 ± 0.04d	0.58 ± 0.01e	1.06 ± 0.03b	0.55 ± 0.03e	0.69 ± 0.01d	0.6 ± 0.01e	1.13 ± 0.01b	1.33 ± 0.01a	0.77 ± 0.01c	0.25	1.53
Hvd	13.08 ± 0.40cd	12.15 ± 0.14d	14.45 ± 0.11c	10.99 ± 0.38e	12.99 ± 0.70cd	12.42 ± 0.36cd	14.98 ± 0.14b	16.35 ± 0.18a	13.42 ± 0.30bc	8.48	17.50
Htd	11.86 ± 0.12cd	11.63 ± 0.13cd	14.60 ± 0.57a	11.80 ± 0.51cd	11.10 ± 0.57d	11.28 ± 0.27d	13.42 ± 0.17bc	14.89 ± 0.23a	12.56 ± 0.29c	7.52	17.04
Cl	5.26 ± 0.21de	4.85 ± 0.04e	8.58 ± 0.10a	4.51±0.22ef	5.91 ± 0.23d	7.43 ± 0.06b	6.90 ± 0.22c	8.51 ± 0.14a	8.22 ± 0.63ab	4.04	13.26
Cw	2.70 ± 0.01cd	2.84 ± 0.06abc	2.85 ± 0.12ab	2.93 ± 0.06a	2.73 ± 0.01bcd	2.69 ± 0.13d	2.95 ± 0.12a	2.78 ± 0.05bc	2.91 ± 0.09a	2.30	3.50
Cl/Hvd	0.40 ± 0.01d	0.38 ± 0.02d	0.59 ± 0.01b	0.41 ± 0.01d	0.43 ± 0.01cd	0.66 ± 0.03a	0.46 ± 0.01cd	0.51 ± 0.02bc	0.56 ± 0.01b	0.36	0.84
Cw/Htd	0.22 ± 0.01c	0.25 ± 0.03a	0.19 ± 0.01d	0.25 ± 0.01ab	0.24 ± 0.01abc	0.22 ± 0.02c	0.22 ± 0.01c	0.18 ± 0.01d	0.23 ± 0.01bc	0.17	0.31
Hsi	1.10 ± 0.02bc	1.06 ± 0.01c	0.99 ± 0.03d	0.94 ± 0.01e	1.16 ± 0.01a	1.10 ± 0.01b	1.11 ± 0.01b	1.09 ± 0.01bc	1.06 ± 0.01bc	0.77	1.35
Hs	0.67 ± 0.01c	0.65 ± 0.02c	0.62 ± 0.01d	0.75 ± 0.01a	0.66 ± 0.01c	0.66 ± 0.01c	0.71 ± 0.01b	0.64 ± 0.01cd	0.64 ± 0.01cd	0.31	0.88
Dmc	6.29 ± 0.02c	6.25 ± 0.19c	5.84 ± 0.06d	6.42 ± 0.19bc	7.08 ± 0.29a	6.65 ± 0.10b	7.05 ± 0.15a	7.32 ± 0.04a	6.38 ± 0.04bc	5.57	7.23
Cfc	0.46 ± 0.01de	0.48 ± 0.02d	0.45 ± 0.01e	0.58 ± 0.01b	0.52 ± 0.02c	0.61 ± 0.01a	0.57±0.02b	0.60 ± 0.01a	0.51 ± 0.01c	0.41	0.66
Ss	1.80 ± 0.01b	1.42 ± 0.02e	1.97 ± 0.01a	1.80 ± 0.02b	1.79 ± 0.01bc	1.72 ± 0.01cd	1.65 ± 0.01d	1.62 ± 0.02d	1.78 ± 0.01bc	1.36	2.03

**Table 4:** Distinctive loci for 01–20 and 01-07-258 compared with the other four sister lines.

Distinctive loci	Locus type	Chromosome No.	Position (cM)	Lines with distinctive loci
*Indel26*	Indel	2	53.4	01–20 and 01-07-258
*Indel488*	Indel	2	54.7	01–20 and 01-07-258
*scaffold29640*	SSR	3	36.0	01–20 and 01-07-258
*Indel64*	Indel	3	37.3	01–20 and 01-07-258
*Indel139*	Indel	5	106.0	01-20
*Indel612*	Indel	8	79.7	01–20 and 01-07-258
*Indel235*	Indel	8	84.7	01–20 and 01-07-258
*Indel353*	Indel	9	58.6	01-07-258

**Table 5:** Distinctive loci and associated agronomic traits in the elite lines 01–20 and 01-07-258.

Loci	Chromosome	Position	Associated traits	LOD	R^2^ (%)	Additive effect
*Indel26*	2	53.4	Lca* 2.1 (2012a)	4.6	8.5	0.4
			Lcb* 2.1 (2012a)	5.98	12.3	−1.08
*Indel488*	2	54.7	—	—	—	—
*scaffold29640*	3	36.0	—	—	—	—
*Indel64*	3	37.3	*Pd 3.2* (2012a)	12.76	19.6	3.42
			*Pd 3.2* (2012s)	10.04	21.2	2.97
			*Ll 3.1* (2012a)	8.74	14.9	1.88
			*Ll 3.1* (2011a)	3.13	6.4	1.29
			*Lw 3.2* (2011a)	5.03	12.7	1.48
			*Pl 3.1* (2012a)	5.93	9.6	1.12
			*Pw 3.1* (2012a)	4.4	7	0.11
			*Hs 3.1* (2012a)	4.92	8.7	−0.04
*Indel139*	5	106.0	—	—		—
*Indel612*	8	79.7	*LcL 8.1* (2011a)	11.64	26.1	−1.04
*Indel235*	8	84.7	*LcL 8.1* (2012s)	5.23	13.9	−0.81
			*HcL 8.1* (2012a)	3.03	6	−0.62
			*Pt 8.1* (2011a)	4.64	11.3	2.01
*Indel353*	9	58.6	Lca* 9.2 (2011a)	4.09	9.5	0.35
			Lca* 9.2 (2012s)	4.49	8	−0.34
			Lcb* 9.1 (2011a)	4.75	9.4	−0.94
			Lcb* 9.2 (2012s)	4.03	9.5	−0.57
			Lcb* 9.2 (2012a)	4.14	8	−0.67
			*Hsi 9.1* (2012s)	3.96	9.6	0.03
			*Cw/Htd 9.1* (2012s)	6.34	13.2	0.01
			*Dmc 9.1* (2012s)	4.4	10.1	0.22
			*Cfc 9.1* (2012s)	6.48	13.9	0.03

**Table 6:** Distinctive loci and the average trait values of the alleles.

Primer	Allele	Associated traits and average values									
		Lca* (2011a)	Lca* (2012s)	Lca* (2012a)	Lcb* (2011a)	Lcb* (2012s)	Lcb* (2012a)	**Dmc (2012s)**	**Cfc (2012s)**	**Hsi (2012s)**	**Cw/Htd (2012s)**
Indel26	a	−6.37	−11.1	−9.16	7.73	15.26	12.37	—	—	—	—
	b	−5.89	−10.53	−8.24	6.92	13.65	10.07	—	—	—	—
Indel353	a	−6.54	−11.2	−9.23	—	—	—	7.05	0.57	1.09	0.25
	b	−5.76	−10.51	−8.31	—	—	—	7.44	0.62	1.15	0.27
		**LcL (2011a)**	**LcL (2012s)**	**LcL (2012a)**	**HcL (2011a)**	**HcL (2012s)**	**HcL (2012a)**	**Pt (2011a)**	**Pt (2012s)**	**Pt (2011a)**	
Indel612	a	33.95	46.5	40.44	—	—	—	—	—	—	
	b	31.99	45.43	39.57	—	—	—	—	—	—	
Indel235	a	34.3	46.84	40.63	60.29	60.44	53.57	61.44	49.71	49.83	
	b	31.93	45.1	39.51	59.04	58.38	52.11	57.72	48.35	48.34	
		**Pd (2012s)**	**Ph (2012s)**	**Ll (2012s)**	**Lw (2012s)**	**Pl (2012a)**	**Pw (2012a)**	**Hs (2012s)**			
Indel64	a	38.59	22.05	20.58	16.95	0	2.80	0.71			
	b	45.24	27.39	23.85	20.12	4.75	3.35	0.71			
