# Supplementary material for: Genome-wide indel/SSR scanning reveals significant loci associated with excellent agronomic traits of a cabbage (Brassica oleracea) elite parental line ‘01–20’
Source: Sci Rep. 2017 Feb 6;7:41696. doi: 10.1038/srep41696 (PMC5292705; doi:10.1038/srep41696)
Supplement: Supplementary Figure 1&2 [file srep41696-s1.pdf]

Genome-wide indel/SSR scanning reveals significant loci associated with excellent agronomic traits of a cabbage (*Brassica oleracea*) elite parental line '01-20'

Honghao Lv[1,§] · Qingbiao Wang[2,§] · Fengqing Han[1] · Xing Liu[1] · Zhiyuan Fang[1] · Limei Yang[1] · Mu Zhuang[1] ·Yumei Liu[1] ·Zhansheng Li[1] ·Yangyong Zhang[1,*]

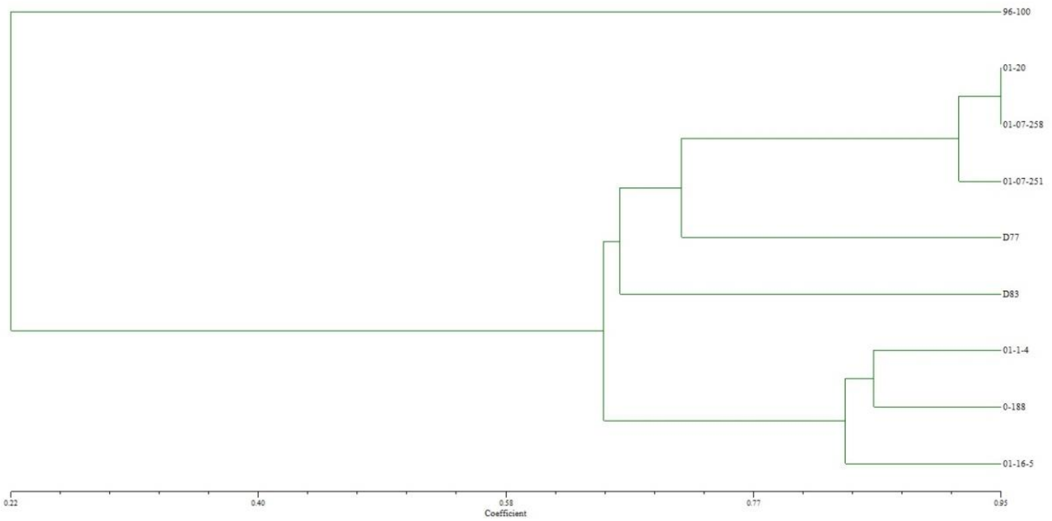

Supplementary Figure 1 Cluster analysis among 96-100, 01-20, five 01-20's sister lines 01-07-258, 01-07-251, 01-1-4, 01-88, 01-16-5, two DH lines D77 and D83.

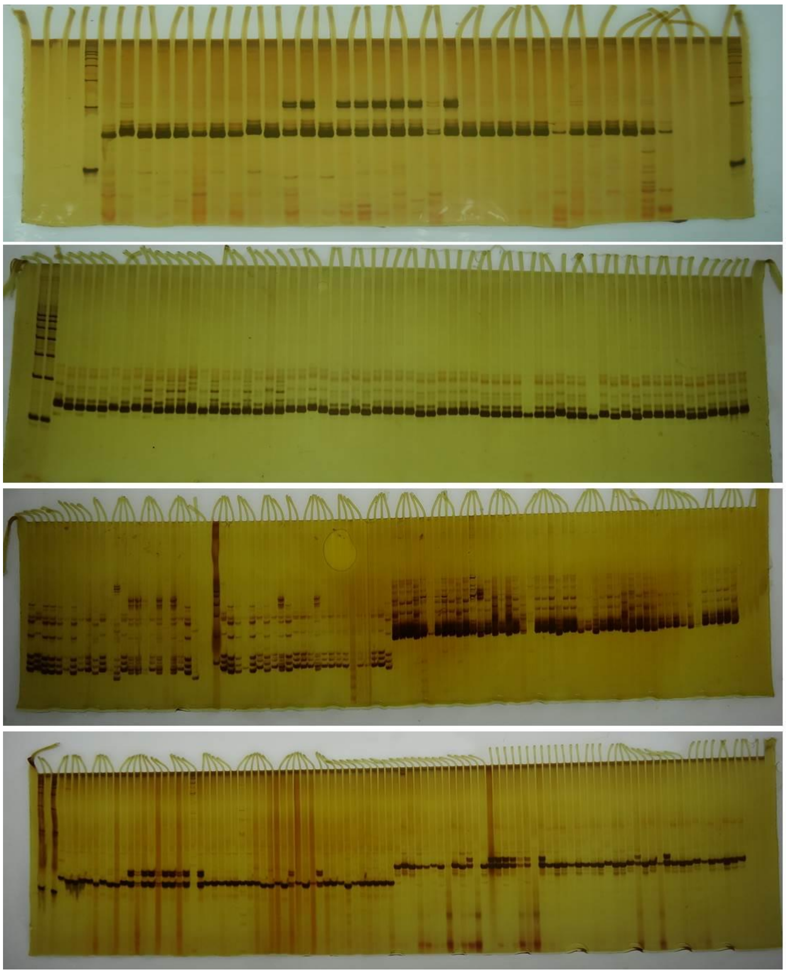

Indel 139

Indel 353

Scaffold 29640

Indel 488

Supplementary Figure 2 Full-length gels for cropped gels in Fig. 3.
